# Draft genome sequence of *Pseudomonas* sp. ER28, a cyclohexane pentanoic acid degrader isolated from oil sands process-affected water from Alberta, Canada

**DOI:** 10.1128/MRA.00651-23

**Published:** 2023-10-24

**Authors:** Kira L. Goff, Jeff Gauthier, Lisa M. Gieg, Irena Kukavica-Ibrulj, Esther Ramos-Padrón, Shawn Lewenza, Roger C. Levesque

**Affiliations:** 1 Department of Immunology and Infectious Diseases, University of Calgary, Alberta, Canada; 2 Faculty of Science and Technology, Athabasca University, Athabasca, Alberta, Canada; 3 Institute of Integrative and Systems Biology, Laval University, Quebec, Canada; 4 Department of Biological Sciences, University of Calgary, Alberta, Canada; DOE Joint Genome Institute, Berkeley, California, USA

**Keywords:** *Pseudomonas*, oil sands tailings, naphthene, cyclohexane pentanoic acid

## Abstract

We report the draft genome sequence of *Pseudomonas* sp. ER28, capable of utilizing the model naphthenic acid, cyclohexane pentanoic acid, as its sole carbon source. It was recovered from oil sands process-affected water containing cyclic and acyclic naphthenic acids. The genome size is 5.7 Mbp, and the G + C content is 60%.

## ANNOUNCEMENT

Industrial activity in oil sands regions results in the production of oil sand process-affected water (OSPW) that must be retained on-site. This complex mixture contains polycyclic aromatic hydrocarbons; benzene, toluene, ethyl benzene, and xylenes; and cyclic and acyclic naphthenic acids (NAs) (1). Bioremediation of these compounds is of great interest.

*Pseudomonas* sp. ER28 is a known degrader of the model NA cyclohexane pentanoic acid (CHPA) via beta oxidation (2). It was collected in 2010 from OSPW from the Athabasca oil sands region of Alberta, Canada, as previously described (2), and transferred into modified Bushnell-Haas medium (2) modified to include CHPA (Sigma-Aldrich) as the sole carbon source to select for organisms capable of its biodegradation.

The isolate was plated on Brain-Heart Infusion (BHI) agar (BD Difco) for 48 h at room temperature (~25°C). Then, 2 mL of BHI broth was inoculated with a pure colony and grown to an OD_600_ of 1.0. Cells were centrifuged (10,000 × *g*) at 4°C for 20 min and DNA extracted using a DNeasy Blood and Tissue Kit (QIAGEN) following the manufacturer’s Gram-negative bacteria protocol. Resultant DNA was quantified via Qubit (dsDNA BR kit, ThermoFisher) and assessed for purity (OD_260_/OD_280_ >1.8) using a NanoDrop 2000 spectrophotometer (ThermoFisher). Total DNA (1 µg) was sheared into fragments of 400–700 bp using the G-tube protocol (Covaris). Paired-end sequencing (2× 300 bp) was done on an Illumina MiSeq apparatus using TruSeq3 PE 2× 300 bp chemistry. Reads were quality-controlled with FastQC (3), trimmed with TRIMMOMATIC version 0.39-2 (4), and assembled using the A5 pipeline version 20150522 (5). Annotation was done with the NCBI Prokaryotic Genome Annotation Pipeline version 6.4 (6) Completeness was assessed with CheckM version 1.2.2 (7). Taxonomy was assigned using either (i) whole-genome *in silico* digital DNA-DNA hybridization via the Type Strain Genome Server (8) or (ii) the average nucleotide identity and phylogeny-based GTDB-Tk (9).

The genome size is 5.70 Mbp at 30× coverage and 100% complete (108 scaffolds, N50 111,151 bp; 5,310 coding DNA sequences, 67 ribosomal RNA genes, 119 transfer RNA genes), with a GC percent value of 62%. All bioinformatics tools were run with default parameters. The closest match was *Pseudomonas putida* NBRC 14164 at only 39.6% (species threshold 70%). Interestingly, GTDB-Tk assigned ER28 to *Pseudomonas hunanensis* slightly above thresholds (0.79% above 95%). This suggests that ER28 is a potential new species related to both *P. putida* and *P. hunanensis*.

## Data Availability

This Whole Genome Shotgun project has been deposited at DDBJ/ENA/GenBank under accession number JARIXT000000000. The version described in this paper is version JARIXT010000000. Raw reads were deposited in the Sequence Read Archive under accession number SRR25168147.
